# Mortality and risk of tuberculosis among people living with HIV in whom TB was initially ruled out

**DOI:** 10.1038/s41598-020-71784-3

**Published:** 2020-09-22

**Authors:** Juan Ignacio García, Edson Mambuque, Dinis Nguenha, Faustino Vilanculo, Charfudin Sacoor, Victor Guillermo Sequera, Manuel Fernández-Quevedo, Maxime Leroux-La Pierre, Helio Chiconela, Luis A. Faife, Durval Respeito, Belén Saavedra, Tacilta Nhampossa, Elisa López-Varela, Alberto L. Garcia-Basteiro

**Affiliations:** 1 TB Group, Population Health Program, Texas Biomedical Research Institute, San Antonio, TX USA; 2 Centro de Investigação em Saude de Manhiça (CISM), Maputo, Mozambique; 3 ISGlobal, Hospital Clínic - Universitat de Barcelona, Barcelona, Spain; 4 Agència de Salut Pública de Barcelona, Barcelona, Spain; 5 Manhiça District Hospital, Ministry of Health, National Tuberculosis Control Program, Maputo, Mozambique; 6 Instituto Nacional de Saúde, Ministério de Saúde, Maputo, Mozambique; 7 Desmond Tutu TB center, Stellenbosch University, Cape Town, South Africa

**Keywords:** Epidemiology, Epidemiology

## Abstract

Tuberculosis (TB) misdiagnosis remains a public health concern, especially among people living with HIV (PLHIV), given the high mortality associated with missed TB diagnoses. The main objective of this study was to describe the all-cause mortality, TB incidence rates and their associated risk factors in a cohort of PLHIV with presumptive TB in whom TB was initially ruled out. We retrospectively followed a cohort of PLHIV with presumptive TB over a 2 year-period in a rural district in Southern Mozambique. During the study period 382 PLHIV were followed-up. Mortality rate was 6.8/100 person-years (PYs) (95% CI 5.2–9.2) and TB incidence rate was 5.4/100 PYs (95% CI 3.9–7.5). Thirty-six percent of deaths and 43% of TB incident cases occurred in the first 12 months of the follow up. Mortality and TB incidence rates in the 2-year period after TB was initially ruled out was very high. The TB diagnostic work-up and linkage to HIV care should be strengthened to decrease TB burden and all-cause mortality among PLHIV with presumptive TB.

## Introduction

Tuberculosis (TB) remains an important public health concern and constitutes the leading cause of death from a single infectious disease agent^1^. According to World Health Organization (WHO)’s estimates, in 2018 there were around 10.0 million of new cases, and 1.5 million deaths attributable to TB. TB morbidity and mortality fuelled by the TB/HIV syndemic^1–3^ is a major global health concern, especially in the African and Asian regions, which account for 75% of the HIV-associated TB cases worldwide^3^. Mozambique is one of the 14 countries included in the high TB, TB/HIV and MDR-TB burden lists, with an estimated TB incidence rate of 551 (356–787) cases per 100,000 in 2018^1^. As it has been estimated that around half of TB cases are not diagnosed (or reported to the health authorities) case detection rates are presumably low^4,5^.

Avoiding TB misdiagnosis is critical to correctly rule in or out TB among TB presumptive individuals. This has special relevance among people living with HIV (PLHIV), who have an increased risk of TB and mortality due to TB^6,7^. TB needs to be accurately diagnosed to reduce both mortality and transmission in the community.

Several studies have shown the increased mortality associated with TB misdiagnosis, in particular, with TB underdiagnosis^8,9^. Although TB is a common cause of death in post-mortem studies conducted in high HIV prevalence settings^10–13^, ascertainment of TB as a cause of death is challenging in most low income countries^14–16^. In addition, PLHIV with clinically diagnosed TB have a higher mortality risk than those with laboratory-confirmed TB^17^ highlighting the importance of improving laboratory-based TB diagnosis in this population. TB mortality is also higher in patients who have already received TB treatment due to a wrong diagnosis, TB sequelae, or missed TB relapse^18,19^.

This study aims to describe the all-cause mortality and TB incidence rates among PLHIV with presumptive TB in which TB was initially ruled out, and to describe sociodemographic risk factors associated with death and incident TB.

## Material and methods

This study is a retrospective review of a cohort of PLHIV presenting to health care facilities with presumptive TB in the district of Manhiça, Mozambique, from August 2013 to August 2014 (one-year period). This analysis includes all PLHIV in whom TB was initially ruled out. Included participants were passively followed-up for a period of 2 years. This analysis was nested in the TOSSE study, a surveillance project which aimed at improving TB diagnosis and reporting in the district of Manhiça^20^.

### Study setting and population

**Figure 1:**
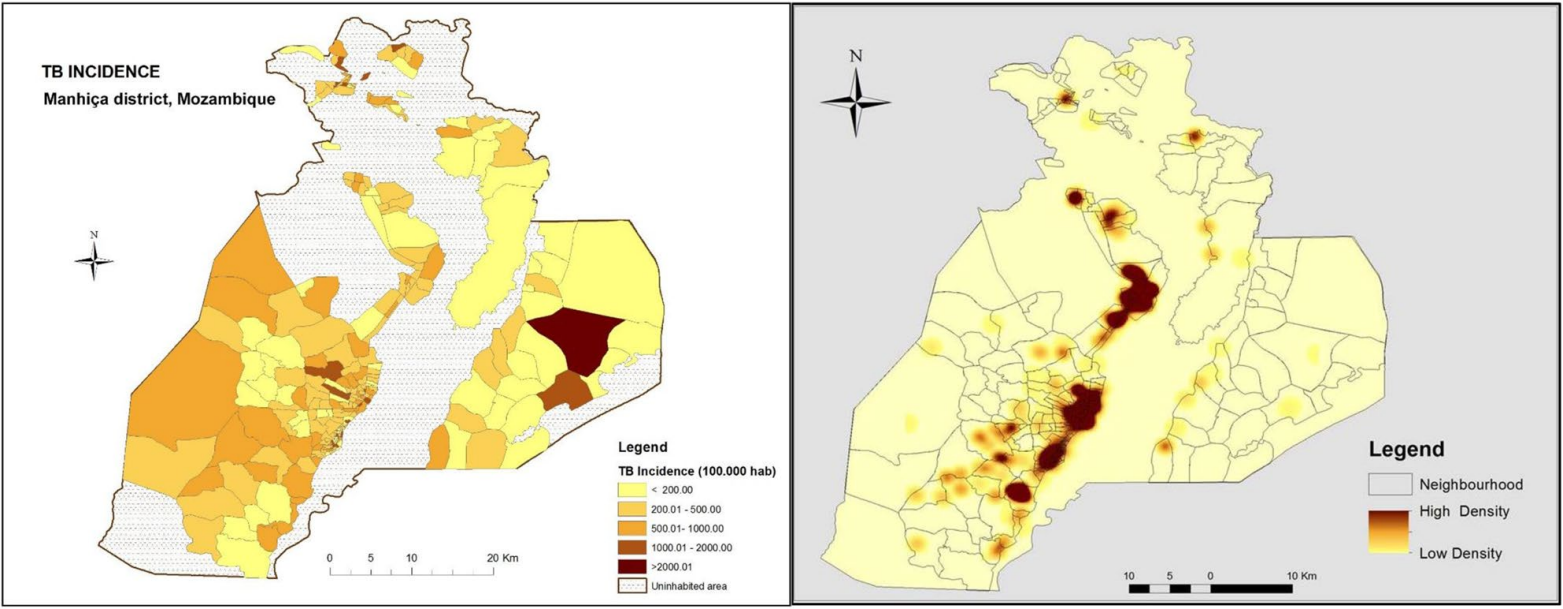
Tuberculosis incidence distribution in the district of Manhiça. Left panel: TB incidence rate per 100,000 population. Right Panel: heat map of TB notifications (2013-2014).

The study was conducted at the Manhiça Health Research Center (*Centro de Investigação em Saúde de Manhiça*, CISM), in the district of Manhiça, Maputo province, southern Mozambique. As per 2016, the estimated population of the district of Manhiça was 186,241 inhabitants living in an area of 2,373 Km^2^. The district has two main Hospitals: Manhiça District Hospital (MDH) and Xinavane Rural Hospital (XRH). This study was conducted in the catchment area of MDH, where approximately 90,000 people lived in 2013. The TB case notification rate was 552 per 100,000 in 2012, although it was much higher among PLHIV^21^. HIV prevalence in the district is high, peaking at 39.9% in individuals aged 18–47, estimated from community based cross-sectional studies^22–24^. TB and HIV treatment is offered free of charge, and for those PLHIV with TB disease, both TB and HIV treatments are provided at the National TB Control Program (PNCT) offices following the so called One Stop Model for TB/HIV^25^. As per national guidelines, isoniazid preventive treatment (IPT) is offered to TB household contacts below 5 years and all PLHIV. The duration of INH preventive therapy is of 6 months and is repeated every 2 years. HIV-positive TB presumptive cases, defined as those presenting at the health care facility of MDH's catchment area with at least one of the following TB symptoms: cough, night sweats, weight loss, fever, regardless of duration and who consented to participate, were enrolled in the TOSSE study (Fig. 1 shows the distribution of TB incidence in the district during the study period).

### General procedures

Enrolled participants answered structured questionnaires and provided information on TB symptoms, previous history of TB, HIV status, demographic characteristics (age, sex, marital status, religion, migrations), socioeconomic status (education level, occupation, income, properties, living conditions) and their knowledge, attitudes and practices concerning TB (mode of transmission, identification of symptoms, treatment and prevention of infection). Additionally, most of them provided a respiratory sample for Xpert MTB/RIF (Xpert) analysis. Presumptive TB cases with negative Xpert were initially treated with broad spectrum antibiotics. If TB symptoms continued and in the absence of a more plausible diagnosis, TB was clinically diagnosed and anti TB treatment was started. This diagnostic work-up usually occurred within one month from the initial visit. Clinical data was collected from all TB cases arising from the presumptive TB cases not diagnosed with TB (medical history, physical examination, laboratory tests, comorbidities and therapeutic information). Participants not diagnosed with TB after the first (suspicion and specimen collection) or second visit (resolution of symptoms with antibiotics if Xpert negative) were retrospectively followed-up for 2 years. Relevant data on key demographic events such as death or migration was obtained from the Health and Demographic Surveillance System (HDSS) run by CISM and described elsewhere^26^. Only study participants with a Perm_id number (unique study participant and household identifier from HDSS) were included in this study; of those study participants who did not provide a Perm_id, a search strategy was used to find potential eligible participants in the HDSS database. We used a combination probabilistic algorithm with name, surname 1, surname 2, age, sex and address. At the time of study initiation, the HDSS covered around 60% of the district population.

Finally, data on TB diagnosis, cART status and TB treatment initiation was collected using records from the PNCT and from the National HIV/AIDS and STI Control Program in the district.

### Laboratory procedures

Sputum samples of presumptive TB cases were obtained in each health care unit and processed for TB diagnosis using Xpert and smear microscopy (Ziehl–Neelsen). Xpert positive samples were then inoculated in liquid culture media (BACTEC MGIT 960-automated; Beckton Dickinson Microbiology Systems, Sparks, MD) and solid media using Lowenstein-Jensen tubes at HDM. Resistance profile was studied through the semiquantitative modified proportion method in liquid medium^27^.

### Data analysis

This analysis is restricted to those HIV positive presumptive TB patients belonging to the HDM catchment area and registered in the HDSS database so vital status or migration could be ascertained.

We used percentages and median and interquartile range (IQR) to describe patients baseline characteristics. Cox proportional hazards regression was used to calculate unadjusted (uHR) and adjusted (aHR) hazard ratios to explore mortality risk factors, TB incidence and the effect of different risk factors for incident TB. Participants were censored at the time of migration. Potential confounding factors used in the model were: age, sex, previous TB episode, family member with a documented TB episode, and BCG vaccination. Those variables showing an association in the univariate analysis with *p* value < 0.2 were tested in the multivariable analysis. The statistical significance for all comparison tests was set at *p* < 0.05. Data analysis was conducted using Stata, version 14 (Stata Corporation, College Station, TX, USA).

### Ethics statement

The study was approved by the CISM’s Internal Scientific Committee, CISM’s Institutional Bioethics Committee for Health (CIBS—Comité Institucional de Bioética para a Saúde) and the National Bioethics Committee for Health (CNB—Comité Nacional de Bioética para a Saúde) of Mozambique. All methods were performed in accordance with the relevant guidelines and regulations. All individuals gave a written informed consent before participation in the study^20^.

## Results

**Figure 2:**
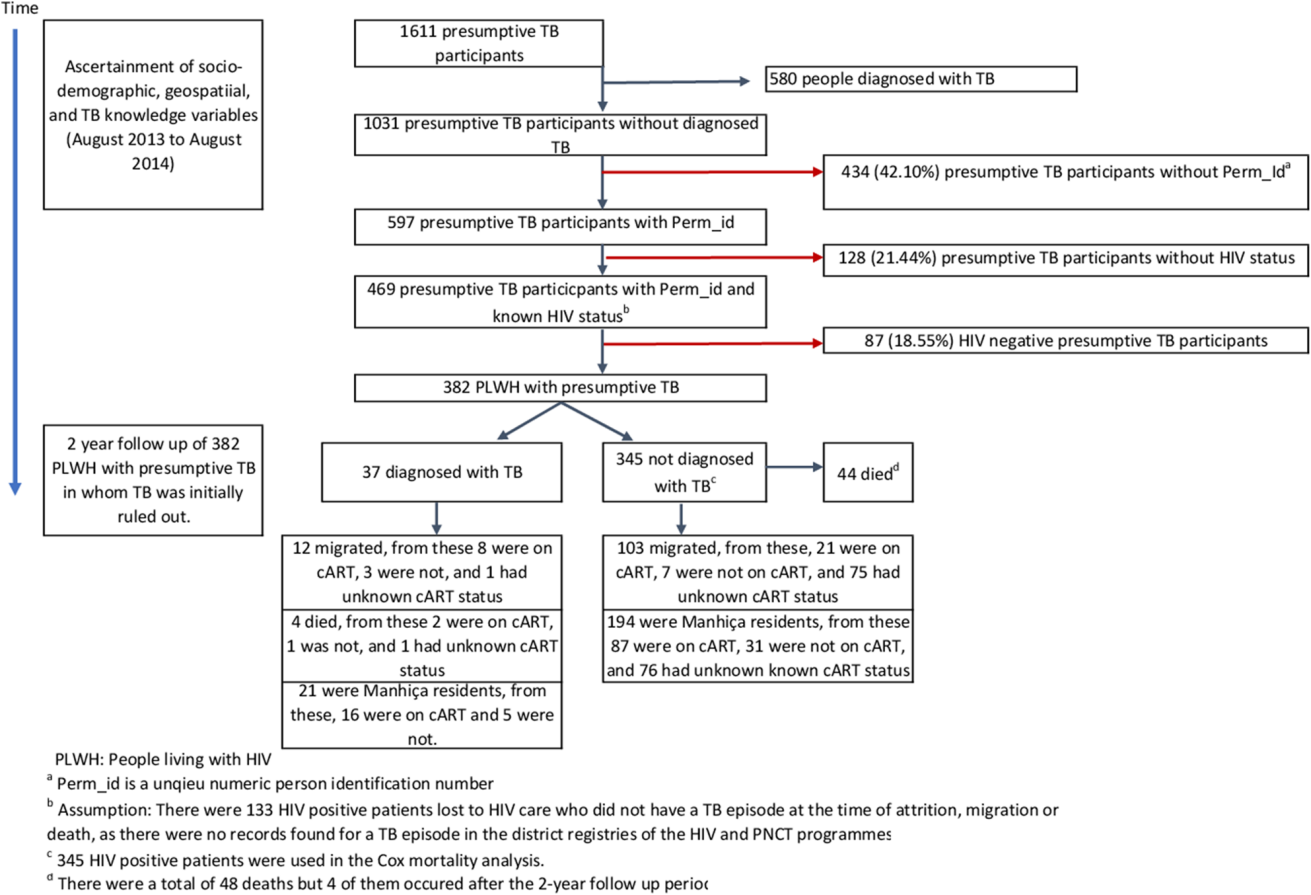
Flowchart of study participants and main endpoints.

From August 2013 to August 2014, a total of 1611 presumptive TB cases were enrolled in the TOSSE study. Of those, 580 cases were initially diagnosed with TB as part of the diagnostic work up: 505 of 580 TB cases (87%) were diagnosed of pulmonary TB and 75 (13%) were diagnosed with extrapulmonary TB. Of those diagnosed with pulmonary TB (505), 321 (63.6%) had a positive Xpert result. Of the 1,031 presumptive TB cases not diagnosed with TB within the first month of the study, 434 (42.1%) could not be found in the HDSS and 128 (21.4%) did not have a known HIV status and were excluded from the analysis. Thus, a total of 382 PLHIV with presumptive TB were included in the analysis (Fig. 2).

Overall, after the 2-year follow-up period of the 382 HIV positive presumptive TB cases, 200 (52.3%) were not on cART or had unknown cART status, 115 (30.1%) migrated out of the area under demographic surveillance, from which 76 (66.1%) had unknown cART status at the time of migration. There were 133 (34.8%) PLHIV lost to HIV care (PLHIV with presumptive TB who did not attend the last HIV care visit scheduled and remained without a follow-up visit within a period of 6 months or more since the last visit attended until the end of the follow-up period). These participants did not have a TB episode at the time of attrition, migration or death, as there were no records found of a TB episode in the district registries of the National HIV and Tuberculosis Control Programs.

The median follow-up period in our cohort was 437.5 days per participant. Characteristics of the 382 HIV-positive presumptive TB cases included in the analysis are shown in Table 1. Overall, 58.9% were females, 33.1% had no occupation, 43.1% had no formal schooling, 75.4% of them had no salary and 75.9% knew that TB was transmitted by coughing.

**Table 1:** Baseline characteristics of 382 PLHIV with presumptive TB participants in whom TB was initially ruled out.

Type of variable	Variable	n^a^	%^b^
Socio-demographic	**Age**		
		371	Mean and IQR: 32.51 (24.51–41.50)
	**Sex**		
	Female	224	58.64
	Male	158	41.36
	**Occupation**		
	Agriculture	143	41.94
	Services	74	21.70
	Student	11	3.23
	No occupation	113	33.14
	**Marital status**		
	Married	61	17.48
	Union	105	30.09
	Widower	27	7.74
	Single	156	44.70
	**Education**		
	None	153	43.22
	Primary school	191	53.95
	Superior	10	2.82
	**Salary type**		
	Regular salary	70	18.32
	Occasional salary	23	6.02
	No salary	289	75.65
	**Worked outside Mozambique**^**c**^		
	Yes	37	10.57
	No	313	89.43
Clinical	**Xpert result**		
	Negative	359	93.98
	Not performed^d^	23	6.02
Habitability and US access	**People living in same house**		
	Number of people		
	from 0 to 3	129	36.34
	from 4 to 6	155	43.66
	7 or more	71	20.00
	**Number of people sleeping in same room**		
	Alone	56	15.73
	from 1 to 3	285	80.06
	4 or more	15	4.21
	**House with windows**		
	Yes	157	44.23
	No	198	55.77
	**Distance in Km from household to HDM**		
	> 15	184	53.80
	From 7.5 to 15	62	18.13
	From 0 to < 7.5	96	28.07
	**Distance in Km from household to closest HCU**		
	From 0 to < 5	309	89.57
	From 5 to 10	32	9.28
	> 10	4	1.16
TB knowledge	**TB transmission knowledge**		
	Cough	271	76.12
	At least 1 misconception	11	3.09
	Cough plus at least 1 misconception	74	20.79
	**TB treatment by traditional medicine can cure TB**		
	Yes	10	3.04
	No	285	86.63
	Do not know	34	10.33

*MDH* Manhiça District Hospital, *HCU* health care unit.

^a^n is sample size of variable specified.

^b^Results are displayed in % if no other specification is provided.

^c^Variable work outside Mozambique is defined as people currently working or who have worked in the past 2 years outside Mozambique.

^d^Those without an Xpert result, TB was ruled out clinically.

### All-cause mortality rate among HIV-positive presumptive TB cases

**Figure 3:**
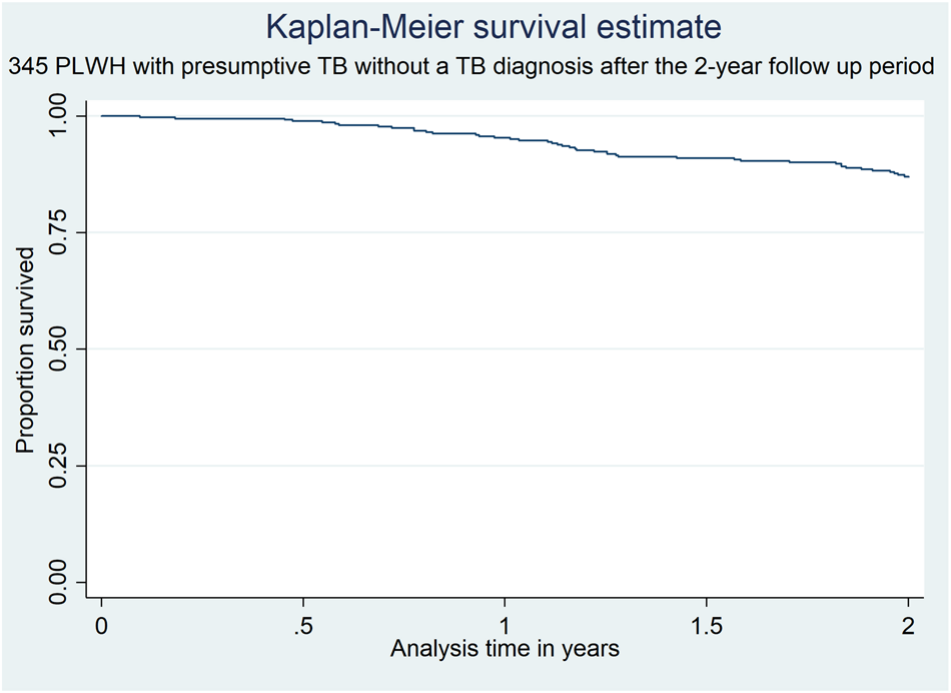
Kaplan Meier survival analysis of presumptive TB cases in whom TB was initially ruled out.

There were 44 deaths (12.7%) among the 345 HIV-positive presumptive TB cases who did not have a documented TB diagnosis after the 2-years of follow-up. The overall mortality rate was 6.8/100 PYs (95% CI: 5.0–9.2). The mortality rate in males and females was 7.2/100 PYs (95% CI: 4.6–11.3) and 6.6/100PYs (95% CI: 4.4–9.7), respectively (Table 2). Over one third of all deaths (16/44, 36.4%) occurred within the first 12 months of follow-up. In our Cox regression model analyzing mortality risk factors, none of the variables studied showed statistical significance associated with dying during the follow-up period (Table 3). Those HIV-positive individuals without formal occupation showed a non-significant aHR of death of 2.1 (95% CI: 0.9–4.7, *p* value = 0.073) compared to those having a registered occupation. Kaplan–Meier survival estimates for the 345 HIV positive presumptive TB cases in whom TB was initially ruled out and did not have a documented TB episode after the 2-year follow-up period are shown in Fig. 3. Thirty- six per cent of deaths occurred within the first 12 months of follow-up.

**Table 2:** Mortality and TB incidence rates among HIV-positive presumptive TB cases included in the study.

	Mortality risk (% and 95% CI)	Mortality rate in PYs (95% CI)	
**Mortality risks and rates (in person-years) in 345 HIV infected presumptive TB participants over a 2-year follow-up period. n = 44 deaths**			
All	12.75 (9.61–16.73)	6.83/100 PYs (5.09–9.18)	
Male	13.29 (8.59–19.99)	7.18/100 PYs (4.58–11.26)	
Female	12.38 (8.47–17.72)	6.59/100 PYs (4.45–9.75)	

	Events = 37		
	TB risk (% and 95% CI)	TB Incidence in PYs (95% CI)	% and 95% CI of laboratory confirmed TB
**TB incidence in PYs in 382 HIV infected presumptive TB participants over a 2-year follow up period**			
All	9.68 (7.09–13.10)	5.41/100 PYs (3.92–7.47)	46 (33–56)
Male	9.49 (5.77–15.22)	5.36/100 PYs (3.23–8.90)	
Female	9.82 (6.53–14.50)	5.44/100 PYs (3.59–8.27)	

*CI:* confidence intervals.

**Table 3:** Unadjusted (uHR) and adjusted (aHR) Cox proportional hazard ratios and 95% confident intervals (CI) for mortality risk factors among 345 HIV-positive presumptive TB participants in whom TB was not documented after 2 years of follow-up.

Type of variable	Variable	44 HIV infected presumptive TB cases died		301 HIV infected presumptive TB cases alive		uHR		aHR^a^	
		n^b^	(%)^c^	n^b^	(%)^c^	(95% CI)	*p* value	(95% CI)	*p* value
Socio-demographic	**Age**	42		281	(%)				
	< 15		7.14		12.46	Ref		Ref	
	15–24		9.52		14.95	1.06 (0.24–4.75)	0.935	1.10 (0.24–4.97)	0.899
	25–34		38.10		28.47	2.19 (0.64–7.53)	0.211	1.94 (0.55–6.77)	0.3
	35–44		11.90		19.93	1.03 (0.25–4.31)	0.967	0.74 (0.16–3.23)	0.695
	45–54		9.52		9.96	1.58 (0.35–7.01)	0.549	1.11 (0.22–5.54)	0.892
	> 55		23.81		14.23	2.62 (0.72–9.54)	0.143	2.65 (0.72–9.71)	0.139
	**Sex**	44		301					
	Female		56.82		58.80	Ref		Ref	
	Male		43.18		41.20	1.09 (0.60–1.99)	0.767	1.10 (0.61–1.99)	0.737
	**Occupation**	38		269					
	Agriculture		34.21		44.24	Ref		Ref	
	Services		26.32		21.19	1.56 (0.69–3.55)	0.285	2.20 (0.83–5.83)	0.112
	Student		0.00		2.97	NA	NA	NA	NA
	No occupation		39.47		31.60	1.59 (0.76–3.35)	0.218	2.10 (0.93–4.71)	0.073
	**Marital status**	40		274					
	Married		20.00		17.88	Ref		Ref	
	Union		25.00		29.93	0.77 (0.30–1.96)	0.583	0.80 (0.30–2.08)	0.646
	Widower		2.50		8.03	0.28 (0.03–2.24)	0.233	0.26 (0.04–1.70)	0.162
	Single		52.50		44.16	1.07 (0.47–2.43)	0.866	1.05 (0.46–2.40)	0.895
	**Number of people living in same house**	40		278					
	From 0 to 3		52.5		34.53	Ref		Ref	
	From 4 to 6		32.5		44.96	0.50 (0.25–1.00)	0.051	0.60 (0.28–1.26)	0.179
	7 or more		15		20.50	0.48 (0.20–1.17)	0.108	0.51 (0.21–1.28)	0.155
Health care access	**Distance in Km from household to MDH**	36		271					
	> 15		27.78		29.89	Ref		Ref	
	From 7.5 to 15		22.22		16.97	1.33 (0.53–3.33)	0.545	0.98 (0.33–2.90)	0.977
	From 0 to < 7.5		50.00		53.14	1.00 (0.46–2.17)	0.999	1.02 (0.44–2.32)	0.97
	**Distance in Km from household to closest HCU**	36		272					
	From 0 to < 5		91.67		90.07	Ref		Ref	
	From 5 to 10		5.56		8.82	0.65 (0.15–2.79)	0.57	0.68 (0.15–3.13)	0.617
	> 10		2.78		1.10	2.05 (0.33–12.68)	0.439	2.08 (0.51–8.51)	0.309
TB transmission and care knowledge	**TB transmission knowledge**								
	(%)	41		280					
	Cough		85.37		75.36	Ref		Ref	
	At leat 1 missconception		0.00		3.21	NA	NA	NA	NA
	Cough plus another TB symptom		14.63		21.43	NA	NA	NA	NA
	**TB treatment by traditional medicine**								
	(%)	39		258					
	No		89.74		86.82	Ref		Ref	
	Yes		0.00		3.49	NA	NA	NA	NA
	Do not know		10.26		9.69	1–05 (0.38–2.92)	0.922	1.11 (0.40–3.13)	0.837

NA: Missing values for variables "Occupation", "TB transmission knowledge" and "TB treatment by traditional medicine" in the multivariable analysis.

MDH: Manhiça District Hospital, HCU: Health care unit.

^a^In the Cox hazard regression analysis, adjustement was performed for the following variables: age, sex, previous episode of TB, having a family member with a previous episode of TB, and BCG vaccination.

^b^n is sample size of variable specified.

^c^Results are displayed in % if no other specification is provided.

### Incidence of TB among HIV-positive presumptive TB cases

Overall, 37 HIV-positive TB cases were diagnosed with TB within the 2-year follow up period (excluding those diagnosed within the first 3 months, which were considered prevalent TB cases: 17 of these (46%) were laboratory-confirmed (Table 2).

Four and 12 out of the 37 incident TB cases died or migrated (10.8 and 32.4% respectively) within the follow-up period. Eleven (29.7%) were not on cART or had unknown cART status.

The TB incidence rate in the cohort was 5.4/100 PYs (95% CI: 3.9–7.5). The TB incidence rates in males and females were 5.4/100 PYs (95% CI: 3.2–8.9) and 5.4/100 PYs (95% CI: 3.6–8.3), respectively. A total of 16 TB cases (16/37, 43.2%) were diagnosed of TB during the first year of follow-up.

In our Cox regression model analyzing risk factors for being diagnosed of TB, being a student (as compared to other occupations) was associated with having a TB diagnosis, aHR: 7.0 (95% CI: 1.9–26.2) *p* value = 0.004. Those HIV-positive presumptive TB cases living from 0 to 7.5 km and 7.5–15 km apart from HDM showed an aHR of being diagnosed of TB of 2.5 (95% CI: 0.9–6.8) and *p* value = 0.065 and of 3.5 (95% CI: 1.1–11.3), and *p* value = 0.040 respectively, compared to those living > 15 km apart from HDM. In addition, those presumptive TB cases reporting no previous episodes of TB in their family members showed a lower risk of TB (aHR of 0.34, 95% CI: 0.12–0.94 and* p* value = 0.038) compared to those reporting previous TB episodes among their relatives. The following variables: age, sex and TB transmission and care knowledge did not show any statistical significance associated with being diagnosed of TB (Table 4).

**Table 4:** Unadjusted (uHR) and adjusted (aHR) Cox proportional hazard ratios and 95% confident intervals (CI) for pulmonary TB disease comparing 37 HIV-positive TB cases and 301 HIV-positive presumptive TB participants without a documented TB diagnosis after 2 years of follow-up.

Type of variable	Variable	37 HIV infected TB cases		301 HIV infected presumptive TB participants		uHR*		aHR^a^	
		n^b^	(%)^c^	n^b^	(%)^c^	(95% CI)	*p* value	(95% CI)	*p* value
Socio-demographic	**Age**	36	(%)	281	(%)				
	< 15		2.78		12.46	Ref		Ref	
	15–24		5.56		14.95	1.60 (0.14–18.06)	0.701	1.41 (0.12–15.80)	0.782
	25–34		36.11		28.47	5.26 (0.67–41.32)	0.114	4.63 (0.60–35.71)	0.142
	35–44		27.78		19.93	5.59 (0.70–44.66)	0.105	5.10 (0.65–39.96)	0.121
	45–54		11.11		9.96	4.49 (0.49–40.89)	0.182	3.89 (0.43–34.96)	0.225
	> 55		16.67		14.23	4.81 (0.56–40.96)	0.15	5.04 (0.60–42.14)	0.136
	**Sex**	37		301					
	Female		59.46		58.8	Ref		Ref	
	Male		40.54		41.2	0.99 (0.51–1.91)	0.983	0.78 (0.36–1.70)	0.54
	**Occupation**	34		269					
	Agriculture		32.35		44.24	Ref		Ref	
	Services		20.59		21.19	1.32 (0.51–3.41)	0.561	1.62 (0.55–4.69)	0.377
	Student		8.82		2.97	3.68 (1.00–13.62)	0.051	7.03 (1.89–26.24)	0.004
	No occupation		38.24		31.60	1.62 (0.73–3.61)	0.236	1.90 (0.83–4.34)	0.127
	**Marital status**	35		314					
	Married		11.43		17.88	Ref		Ref	
	Union		37.14		29.93	1.92 (0.63–5.87)	0.25	2.15 (0.65–7.08)	0.209
	Widower		11.43		8.03	2.08 (0.53–8.12)	0.291	1.53 (0.33–7.13)	0.585
	Single		40.00		44.16	1.44 (0.48–4.37)	0.511	1.85 (0.58–5.96)	0.3
Health care access	**Distance in km from household to HDM**	35		307					
	> 15		14.29		29.89	Ref		Ref	
	From 7.5 to 15		22.86		16.97	237 (0.90–6.22)	0.08	3.46 (1.06–11.31)	0.04
	From 0 to < 7.5		62.86		53.14	2.61 (0.86–7.89)	0.089	2.53 (0.94–6.78)	0.065
	**Distance in km from household to closest HCU**	37		308					
	From 0 to < 5		83.78		90.07	Ref		Ref	
	From 5 to 10		16.22		8.82	1.83 (0.78–4.29)	0.166	1.91 (0.82–4.45)	0.133
	> 10		0		1.1	NA	NA	NA	NA
TB transmission and care knowledge	**TB transmission knowledge**								
	(%)	35		321					
	Cough		71.43		75.36	Ref		Ref	
	At leat 1 missconception		5.71		3.21	1.86 (0.44–7.78)	0.394	1.67 (0.42–6.63)	0.464
	Cough plus another TB symptom		22.86		21.43	1.10 (0.50–2.43)	0.81	0.83 (0.35–2.00)	0.686
	**TB treatment by traditional medicine**								
	(%)	32		297					
	No		81.25		86.82	Ref		Ref	
	Yes		3.13		3.49	1.00 (0.14–6.94)	1	0.44 (0.03–5.27)	0.514
	Do not know		15.63		9.69	1.70 (0.66–4.38)	0.274	1.72 (0.71–4.21)	0.232

NA: Missing values for variables "Occupation", "TB transmission knowledge" and "TB treatment by traditional medicine" in the multivariable analysis.

*MDH* Manhiça Dsitrict Hospital, *HCU* Health care unit.

^a^In the Cox hazard regression analysis, adjustement was performed for the following variables: age, sex, previous episode of TB, having a family member with a previous episode of TB, and BCG vaccination.

^b^n is sample size of variable specified.

^c^Results are displayed in % if no other specification is provided.

## Discussion

This study is one of the few analyses of important outcomes (all-cause mortality, TB incidence) in a cohort of PLHIV with presumptive TB in whom TB was initially ruled out using routine clinical diagnosis and laboratory procedures. Overall, our results show a high all-cause mortality and TB incidence rate (6.8/100 PYs and 5.4/100PYs respectively), which should prompt a revision of TB diagnostic algorithms and clinical follow-up of PLHIV with presumptive TB.

Surprisingly, our all-cause mortality risk of 12.7% is higher than the crude mortality risk of 5–10% in HIV-positive patients with TB disease under cART in a cohort study in Tanzania^28^, taking into account that 38 (11%) and 151 (43.8%) of our study participants were not on cART or had unknown cART status, respectively. Although we could not document the final cause of death, it is likely that TB (despite initial rule-out), was the major single contributor to mortality in our cohort. A recent meta-analysis shows that TB accounts for approximately 40% of HIV related deaths, and almost 50% remain undiagnosed of TB at the time of death^29^.

Our analysis on potential risk factors for mortality does not identify any key associated factor. However, published data show lower mortality with increasing number of family members living in the same household, as suggested previously by the fact that family members' awareness of TB and HIV symptoms, treatment and care could also deal with complex stigma and discrimination perceptions^30,31^, making family support a positive influence in seeking health care access^32,33^.

A high percentage (29.8%) of migration was observed in those 345 HIV-positive presumptive TB cases in whom TB was not documented during the 2-year follow-up period. Public health systems in sub-Saharan settings are not strengthened enough to properly manage the challenges associated with migration patterns among PLHIV, which might reduce retention in HIV care, increase HIV drug resistance and thus, HIV associated mortality^34^. Socio-demographic factors, internal and external migration, fragile health care systems and TB and HIV transfers out are key elements likely to hinder TB/HIV treatment and retention in care, as previously suggested^35–37^, and all of these factors contribute to higher mortality. Therefore, considering that in our study 66% of the PLHIV who migrated were not on cART or had unknown cART status at the time of migration, it is likely that our mortality and TB incidence rates are an underestimation of the true rates.

TB incidence rates found in our study are very high (5.41/100 PYs), higher than the TB incidence rates in the general HIV-positive population in our setting^4^. Our TB incidence is almost 10 times higher than the TB incidence rate found in a recent retrospective cohort analysis in Nigeria among HIV-positive patients initiating cART (0.57/100PYs)^38^ and in a study in rural Tanzania (1.7/100PYs)^39^. Nevertheless, our figures are similar to those from a recent prospective cohort study, in South Africa, which reported a TB incidence rate of 5.4/100PYs in HIV-positive household contacts of TB patients^40^. These high TB rates could partially be explained due to PLHIV having advanced HIV disease, poor diagnostic work-up and high numbers of missed TB cases, many of them being missed during the first visit to the health facilities. Taking into account the low case detection rate in Mozambique, under or misdiagnosis is a likely event in HIV-positive patients with TB symptoms. TB misdiagnosis has been reported to occur in rural health care centers and overcrowded reference health centers run by health care workers with limited training^15,41,42^. A recent cohort study conducted in Malawi, which assessed presumptive TB adults with chronic cough after 12 months of the initial visit found that TB was diagnosed in 10% of these patients and the mortality risk was of 4.1%^43^. Another prospective study in Zambia found that in those inpatients without presumptive TB (i.e. without presence of cough of 2 or more weeks at admission)  but who were able to provide a sputum sample, 19/133 (14.3%) were HIV-positive and 7/118 (5.9%) had culture-confirmed TB^10^. Thus, given the low case detection rate in the Mozambique and the lack of active case finding strategies during the study period, misdiagnosis could have been due to poor diagnostic work-up and the intrinsic challenges to diagnose TB in HIV-positive patients, who are frequently paucibacillary and often asymptomatic.

The population in our setting is highly mobile, (29.9% of our cohort had migrated within the 2-year follow-up period) with many migrants travelling for work opportunities to other provinces of Mozambique, but especially to South Africa^26^. These migration patterns have strong implications for TB and HIV prevention and control. Other factors such as HIV and TB stigma, discrimination, gender inequalities and sexual violence, hinders health seeking behaviors as described previously^32^.

In our cohort, being a student was associated with a TB diagnosis during follow-up with an aHR of 7.28 (95% CI: 2.0–27.1; *p* value = 0.003) compared to other occupations. We believe this is likely associated with increased detection rather than with increased risk of TB. Despite the lack of specific TB screening programs within schools, they are generally close to health care centres. Some schools are often part of health prevention campaigns and might be more likely to be aware of TB prevention and control measures, thus being more likely to access health care, be screened, referred and diagnosed with TB if they experience an episode of disease.

Students diagnosed with TB showed a median age of 16.5 IQR (8.54–17.42), and 66.7% were in the range of 15–24 years old. These results are in concordance with reported data from Nigeria where young people between 25 and 29 years were more likely to have HIV-associated TB compared to people above 35 years of age^30^. In addition, HIV-positive TB cases living from 0–7.5 km to 7.5–15 km apart from HDM showed an aHR of TB diagnosis of 2.5 (95% CI: 0.9–6.8 and *p* value = 0.065) and of 3.5 (95% CI: 1.1–11.3, and *p* value = 0.040) respectively. These data suggest that people living closer to health care centers might be more likely to be diagnosed with TB because proximity facilitates health care seeking behaviors, HIV retention in care^44,45^ and decreases TB diagnostic delays^46,47^. On the other hand, we could also speculate that people living far away from health care centers may have more TB burden due to lack of access to TB care and therefore TB is less likely to be diagnosed and thus death is more likely to occur^9^.

### Limitations

Our study had several limitations. First, the proportion of patients that were found on the HDSS database (around 42%) was only a proportion of the expected amount (60%) and therefore selection bias might have occured. This is due to difficulties of matching data collected in different databases, where some variables (age, address) and names are provided differently. However, we believe the population under HDSS does not differ significantly from that not under HDSS; the areas included in the HDSS are not closer to any particular health facility so we believe there is no geographic bias in the inclusion of patients from the HDSS compared to those outside the HDSS. In addition, we did not have the HIV status of all presumptive TB cases, thus there is the possibility of selection bias if the characteristics of included patients is different from the actual population of PLHIV with presumptive TB. Nonetheless, we believe the chances of matching study participants with the HDSS database are random and the proportion of presumptive cases without HIV status was relatively low (21.4%). Second, the final sample size was small, and the limited number of deaths and TB cases made it difficult to find factors associated with the TB mortality or incident TB. Likewise, the high rate of migration led to data censoring and uncertainty about mortality and disease outcomes among migrants. Third, we could not collect data on many interesting variables, such as adherence to HIV care recommendations, cause of death, TB laboratory confirmation during follow-up or other visits to the health care facility, which restricts the data analysis and interpretation. Fourth, given the imperfect characterization of the TB presumptive phenotype, it is impossible to elucidate which deaths could be related to TB or which “new” TB episodes were part of the episode causing the initial visit. It is likely that there is a real overestimation of TB incidence among PLHIV if TB disease had been properly ruled out. Nonetheless, the low case detection and passive disease surveillance system may contribute to an underdiagnosis of the true disease burden among PLHIV.

In conclusion, TB incidence and mortality in PLHIV with presumptive TB in whom TB was initially ruled-out is very high, much higher than in the general HIV-positive population in the district of Manhiça. These findings highlight the importance of improving TB diagnostic algorithms and further efforts for patient retention on HIV care.
